# *White Spot Syndrome Virus* (WSSV) Inhibits Hippo Signaling and Activates Yki To Promote Its Infection in Penaeus vannamei

**DOI:** 10.1128/spectrum.02363-22

**Published:** 2022-12-08

**Authors:** Linwei Yang, Zi-Ang Wang, Ran Geng, Hengwei Deng, Shengwen Niu, Hongliang Zuo, Shaoping Weng, Jianguo He, Xiaopeng Xu

**Affiliations:** a State Key Laboratory of Biocontrol, School of Life Sciences, Sun Yat-sen University, Guangzhou, People’s Republic of China; b Southern Marine Science and Engineering Guangdong Laboratory (Zhuhai), Zhuhai, People’s Republic of China; c Institute of Aquatic Economic Animals and Guangdong Province Key Laboratory for Aquatic Economic Animals, Sun Yat-sen University, Guangzhou, People’s Republic of China; University of Prince Edward Island

**Keywords:** *White spot syndrome virus*, shrimp, *Penaeus vannamei*, Yki, Hippo, Wts, Dorsal, apoptosis

## Abstract

*White spot syndrome virus* (WSSV) is a serious threat to shrimp aquaculture, especially Pacific white shrimp, Penaeus vannamei, the most farmed shrimp in the world. Activation of the Hippo-Yki signaling pathway, characterized by the intracellular Hippo-Wts kinase cascade reactions and the phosphorylation and cytoplasmic retention of Yki, is widely involved in various life activities. The current work established the fundamental structure and signal transduction profile of the Hippo-Yki pathway in *P. vannamei* and further investigated its role in viral infection. We demonstrated that WSSV promoted the dephosphorylation and nuclear translocation of Yki, suggesting that Hippo signaling is impaired and Yki is activated after WSSV infection in shrimp. *In vivo*, *Yki* gene silencing suppressed WSSV infection, while *Hippo* and *Wts* silencing promoted it, indicating a positive role of Hippo signaling in antiviral response. Further analyses showed that Yki suppressed Dorsal pathway activation and inhibited hemocyte apoptosis in WSSV-infected shrimp, while Hippo and Wts showed opposite effects, which contributed to the role of Hippo signaling in WSSV infection. Therefore, the current study suggests that WSSV annexes Yki to favor its infection in shrimp by inhibiting Hippo signaling.

**IMPORTANCE** *White spot syndrome virus* (WSSV) is one of the most harmful viral pathogens to shrimp. The pathological mechanism of WSSV infection remains unclear to date. The Hippo-Yki signaling pathway is important for various biological processes and is extensively involved in mammalian immunity, but little is known about its role in infectious diseases in invertebrates. Based on revealing the fundamental structure of the shrimp Hippo pathway, this study investigated its implication in the pathogenesis of WSSV disease. We demonstrated that WSSV enhanced Yki activation by inhibiting Hippo signaling in shrimp. The activated Yki promoted WSSV infection by inhibiting hemocyte apoptosis and suppressing the activation of Dorsal, an NF-κB family member in shrimp that is critical for regulating antiviral response. Therefore, this study suggests that WSSV can hijack the Hippo-Yki signaling pathway to favor its infection in shrimp.

## INTRODUCTION

*White spot syndrome virus* (WSSV), the only member of the *Whispovirus* genus, family Nimaviridae, has a large double-stranded DNA genome and is one of the most harmful viral pathogens to crustaceans (1, 2). WSSV infection leads to white spot syndrome (WSS) and mass mortality of crustaceans, potentially threatening the marine ecology and causing great economic losses to the shrimp farming industry (3, 4). Pacific white shrimp, Penaeus vannamei (syn., Litopenaeus vannamei), currently the most cultured shrimp in the world, is a representative species of the subphylum Crustacea with an important evolutionary status (5). In recent years, *P. vannamei* farming has been heavily threatened by WSSV. Understanding the pathogenic mechanism of WSSV infection is the key to controlling WSS in aquaculture.

The Hippo pathway, originally discovered in Drosophila melanogaster, is evolutionarily conserved in animals and acts as a key player in organ development and regeneration by regulating cell growth, survival, mobility, proliferation, and differentiation (6–8). In *Drosophila*, the kinases Hippo and Warts (Wts), corresponding to mammalian MstI/2 (also called STK4/STK3) and Lats1/2, respectively, and the downstream transcriptional coactivator Yorkie (Yki), corresponding to mammalian Yes-associated protein (YAP), constitute the fundamental structure of the canonical Hippo pathway (9–11). When Hippo signaling is not activated, the unphosphorylated Yki is located in the nucleus and interacts with the transcription factor Scalloped (sd) to activate expression of multiple target genes, such as the proproliferative gene cyclin E and the antiapoptotic gene DIAP1 (10, 12). Upon cell stress, Hippo is activated to promote the phosphorylation of Wts, which further phosphorylates Yki to inhibit its nuclear translocation, resulting in promotion of cell death and arrest of cell proliferation (13, 14). The Hippo-Yki pathway in mammals shares the same pattern of signal transduction and Yki activation/inactivation with *Drosophila* (15).

Dysregulation of the Hippo pathway leads to aberrant tissue growth and cancer occurrence and development, attracting more and more research attention currently (16–18). In mammals, accumulating clues have pointed to the role of Hippo signaling in immunity (19, 20). The Hippo-Yki signaling regulates the production of proinflammatory cytokines and metabolism, intracellular homeostasis, maturation, migration, and infiltration of innate immune cells (21, 22). By cross talking with other immune pathways, such as the NF-κB, JAK-STAT, and mitogen-activated protein kinase (MAPK) signaling cascades, the Hippo pathway is also involved in the pathological processes of inflammation, cancer, and many other diseases (23–25). However, the physiological roles of Hippo-Yki signaling in the innate immune response and the exact underlying mechanisms are still poorly understood. As Hippo signaling is highly conserved across higher and lower animals, studying its function in invertebrates could help to understand its role in the innate immune system from an evolutionary perspective.

The current study analyzed the fundamental framework and operational mechanism of Hippo-Yki signaling in shrimp and explored its role in WSSV infection. We showed that Hippo signaling was inhibited in WSSV-infected shrimp, leading to Yki overactivation, which in turn facilitated WSSV infection. This finding may help to understand the antiviral mechanism of shrimp and the pathogenesis of WSSV.

## RESULTS

### Expression analysis of Hippo-Yki pathway components.

**FIG 1:**
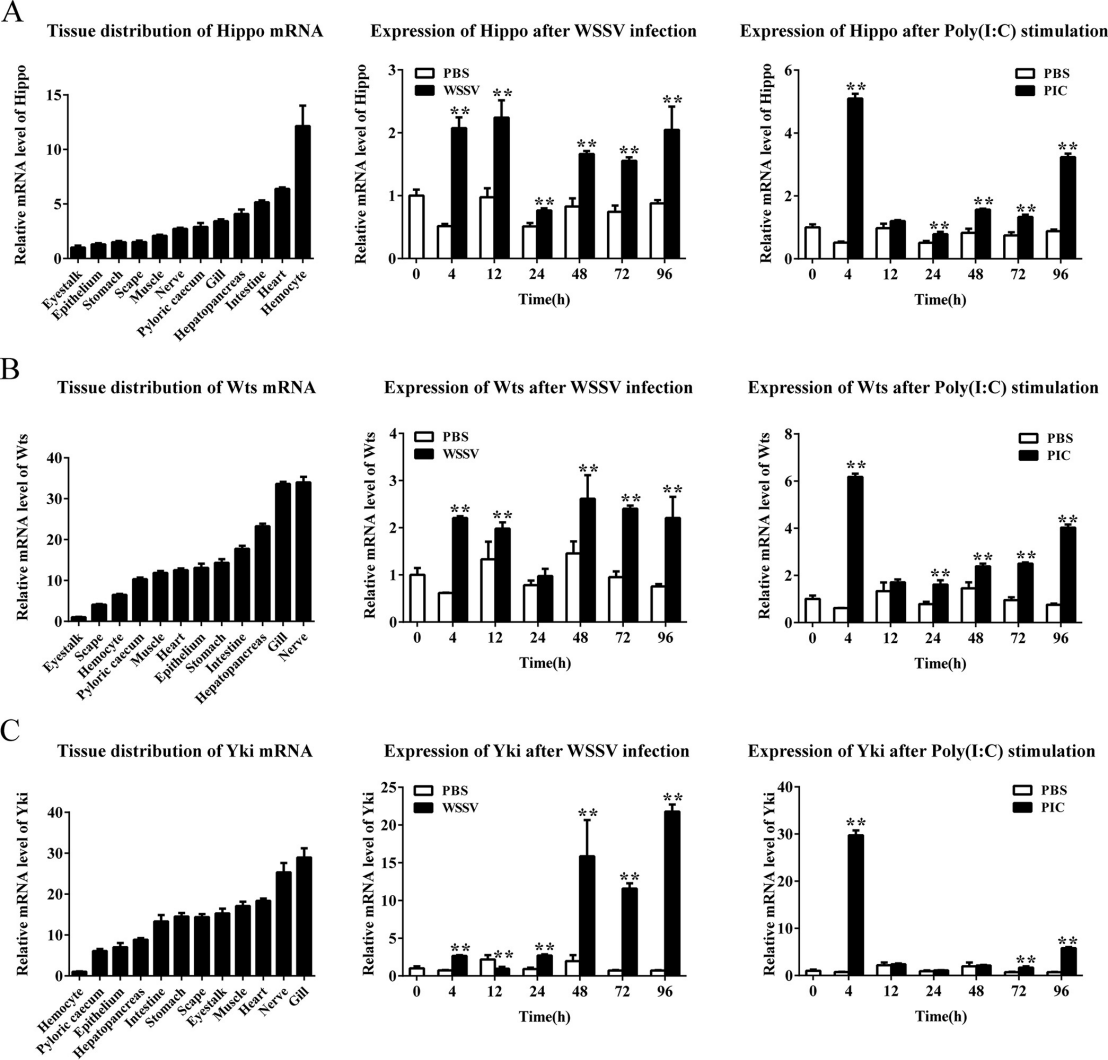
Expression of the Hippo pathway components in shrimp. (A to C) The mRNA distribution in shrimp tissues and the expression profiles of *Hippo* (A), *Wts* (B), and *Yki* (C) in WSSV- and poly(I·C)-stimulated hemocytes were investigated using qPCR. The data shown are representative of three experiments and presented as means ± SD of four detections. *, *P < *0.05; **, *P < *0.01; by Mann-Whitney test.

The mRNA levels of *Hippo*, *Wts*, and *Yki*, the three key component genes of the Hippo-Yki pathway, in *P. vannamei* tissues were analyzed using quantitative PCR (qPCR) (Fig. 1A, C, and E). The expression level of *Hippo* was highest in hemocytes, and had little difference in most other tissues (Fig. 1A). The lowest expression of *Hippo* was observed in the eyestalk. In contrast, *Wts* and *Yki* were most highly expressed in gills and nerves, but both were low in hemocytes (Fig. 1C and E). Similar to *Hippo*, *Wts* has the lowest expression in the eyestalk, while *Yki* expression is lowest in hemocytes. The inconsistent expression levels of these three components suggest that the degree of inhibition of *Yki* by *Hippo* and *Wts* in different tissues may be different, which deserves further investigation. Their expression profiles in hemocytes after viral infection were investigated by qPCR. After WSSV infection, the expression of *Hippo* and *Wts* showed similar upward trends, both with the highest increases less than 4-fold compared to the controls. After stimulation with the synthetic viral double-stranded RNA (dsRNA) analogue poly(I·C), the expression trends of the two kinases were also similar, both reaching the highest levels at 4 and 96 h postinfection (hpi). In contrast, the expression of *Yki* increased more significantly after WSSV and poly(I·C) stimulations. The mRNA levels of *Yki* at 96 h post-WSSV infection and 4 h post-poly (I·C) stimulation reached 30.8- and 40.5-fold those of the controls, respectively.

### Activation profile of the shrimp Hippo-Yki pathway.

**FIG 2:**
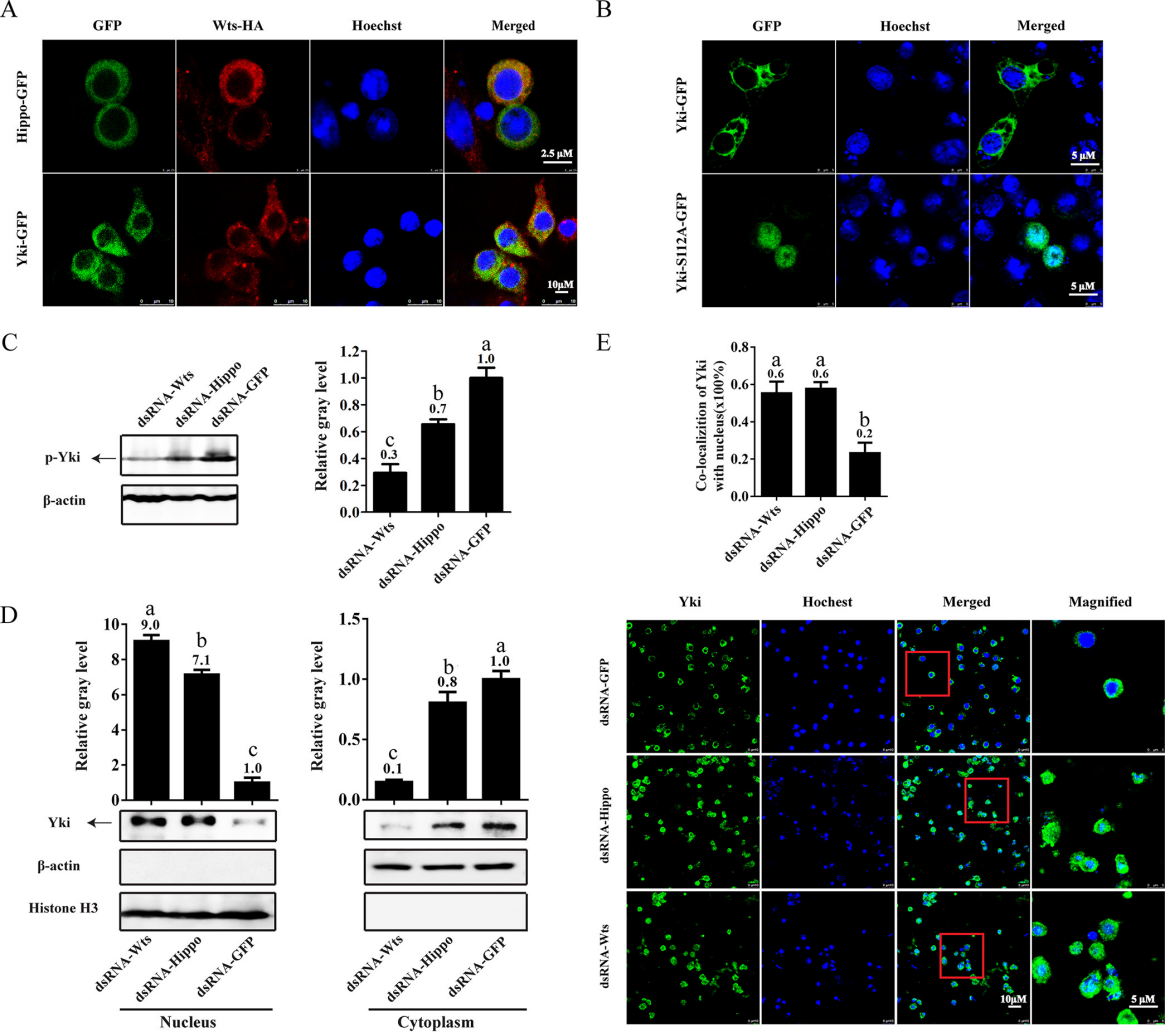
Activation profiles of Hippo signaling in shrimp. (A and B) Subcellular localization of shrimp Hippo, Wts, and Yki in transfected S2 cells. The GFP-tagged Hippo, Yki, and Yki-S112A mutant were directly visualized (green), and the HA-tagged Wts was detected by immunofluorescence (red). The nucleus was stained with Hoechst 33342 (blue). (C and D) The phosphorylation and nuclear-translocation of Yki regulated by Hippo and Wts *in vivo*. Hemocytes from *Hippo*- and *Wts*-silenced shrimp were analyzed using Western blotting. The gray values of the bands of phosphorylated Yki (p-Yki) in whole cells (C) and Yki in the cytoplasm (D, right panel) were normalized to β-actin, and those of Yki in the nucleus (D, left panel) were normalized to histone H3. (E) Immunofluorescent analysis of the nuclear-translocation of Yki in hemocytes after silencing of *Hippo* and *Wts*. The proportion of Yki nuclear-location cells in total *Yki*-expressing cells (green) was calculated in three random visual fields (the above histogram). Results are representative of three independent experiments. Values with different letters indicate significant differences as determine by one-way ANOVA followed by Dunnett’s *post hoc* test (**, *P* < 0.01).

In transfected *Drosophila* S2 cells, the ectopically expressed *P. vannamei* Hippo and Yki proteins were mainly present in the cytoplasm and colocalized with Wts (Fig. 2A). Without coexpression with shrimp Wts, Yki was also located in the cytoplasm, indicating that it could be prevented from entering the nucleus by endogenous Hippo and Wts in S2 cells (Fig. 2B). The 112th serine (Ser112) of *P. vannamei* Yki corresponds to the Ser127 of human Yki, the phosphorylation of which inhibits the nuclear translocation of Yki. Mutation of this site in shrimp Yki was achieved by replacing the serine (S) with alanine (A) to generate the Yki-S112A mutant. We observed that Yki-S112A protein was mainly located in the nucleus, suggesting that this site is also important for the regulation of Yki nuclear translocation in shrimp (Fig. 2B). Western blot analysis further demonstrated that the phosphorylation of Yki was inhibited in hemocytes of *Hippo*- and *Wts*-silenced shrimp (Fig. 2C). Consistent with this, both Western blot and immunofluorescent assays showed that after silencing of *Hippo* and *Wts*, the translocation of Yki from cytoplasm to nucleus in hemocytes was significantly promoted compared with the control (Fig. 2D and E).

### Hippo signaling inhibits Dorsal activation in shrimp.

**FIG 3:**
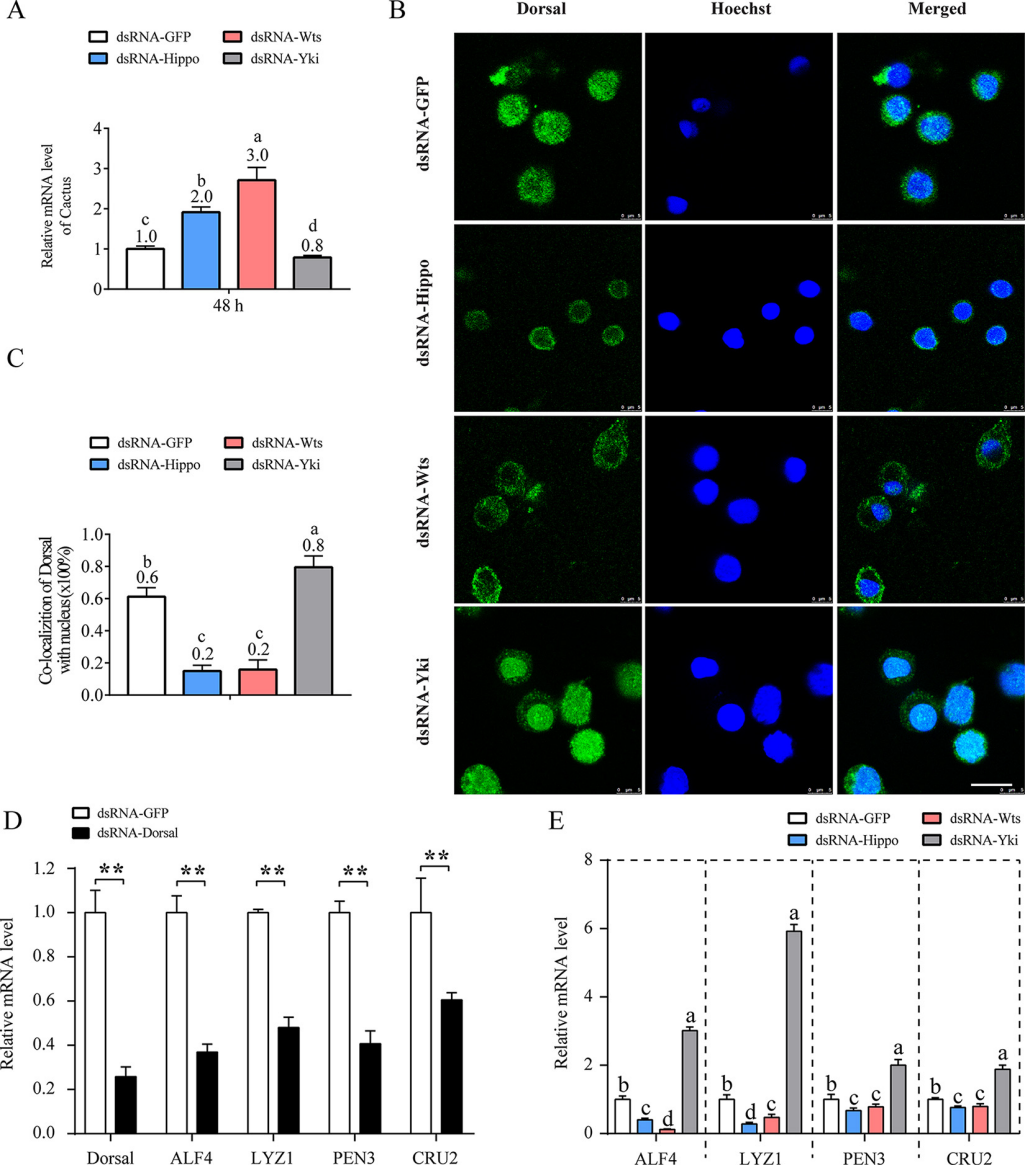
Inhibition of Dorsal by Hippo signaling. (A) qPCR analysis of the expression of Cactus in hemocytes after silencing of *Hippo*, *Wts*, and *Yki*. (B) Nuclear translocation of Dorsal (green) in hemocytes from *Hippo*-, *Wts*-, and *Yki*-silenced shrimp analyzed by immunofluorescence. (C) The proportion of Dorsal nuclear-location cells in total cells was calculated in three random visual fields. (D and E) Expression of the Dorsal pathway downstream genes *ALF4*, *PEN3*, *CRU2*, and *LYZ1* in hemocytes after silencing of *Dorsal* (D) and *Hippo*, *Wts*, and *Yki* (E) was detected by qPCR. **, *P < *0.01 by one way ANOVA with Dunnett's *post hoc* test (A, C, and E) and Mann-Whitney test (D).

The Dorsal pathway plays a central role in shrimp immunity (26). The regulatory effect of the Hippo pathway on Dorsal activation was therefore investigated. Degradation of the Dorsal inhibitor Cactus and the following nuclear translocation of Dorsal are hallmarks of Dorsal pathway activation (27). Compared with the control, the expression of *Cactus* was increased after *Hippo* and *Wts* silencing but decreased after *Yki* silencing (Fig. 3A). Consistent with these results, the nuclear translocation of Dorsal was suppressed in hemocytes of *Hippo*- and *Wts*-silenced shrimp but was promoted in hemocytes of *Yki*-silenced shrimp (Fig. 3B and C). Moreover, the regulation of the Dorsal pathway downstream effector genes antilipopolysaccharide factor 4 (*ALF4*), penaeidin 3 (*PEN3*), Crustin 2 (*CRU2*), and lysozyme 1 (*LYZ1*) by Dorsal was confirmed by qPCR, which showed that their expression was downregulated in Dorsal-silenced shrimp (Fig. 3D). In contrast, their expression was significantly reduced upon silencing of *Hippo* and *Wts* but upregulated upon silencing of *Yki* (Fig. 3E). This confirms that the activation of Hippo signaling can facilitate the activation of the Dorsal pathway in shrimp.

### Suppression of Hippo-Yki signaling by immune stimulation.

**FIG 4:**
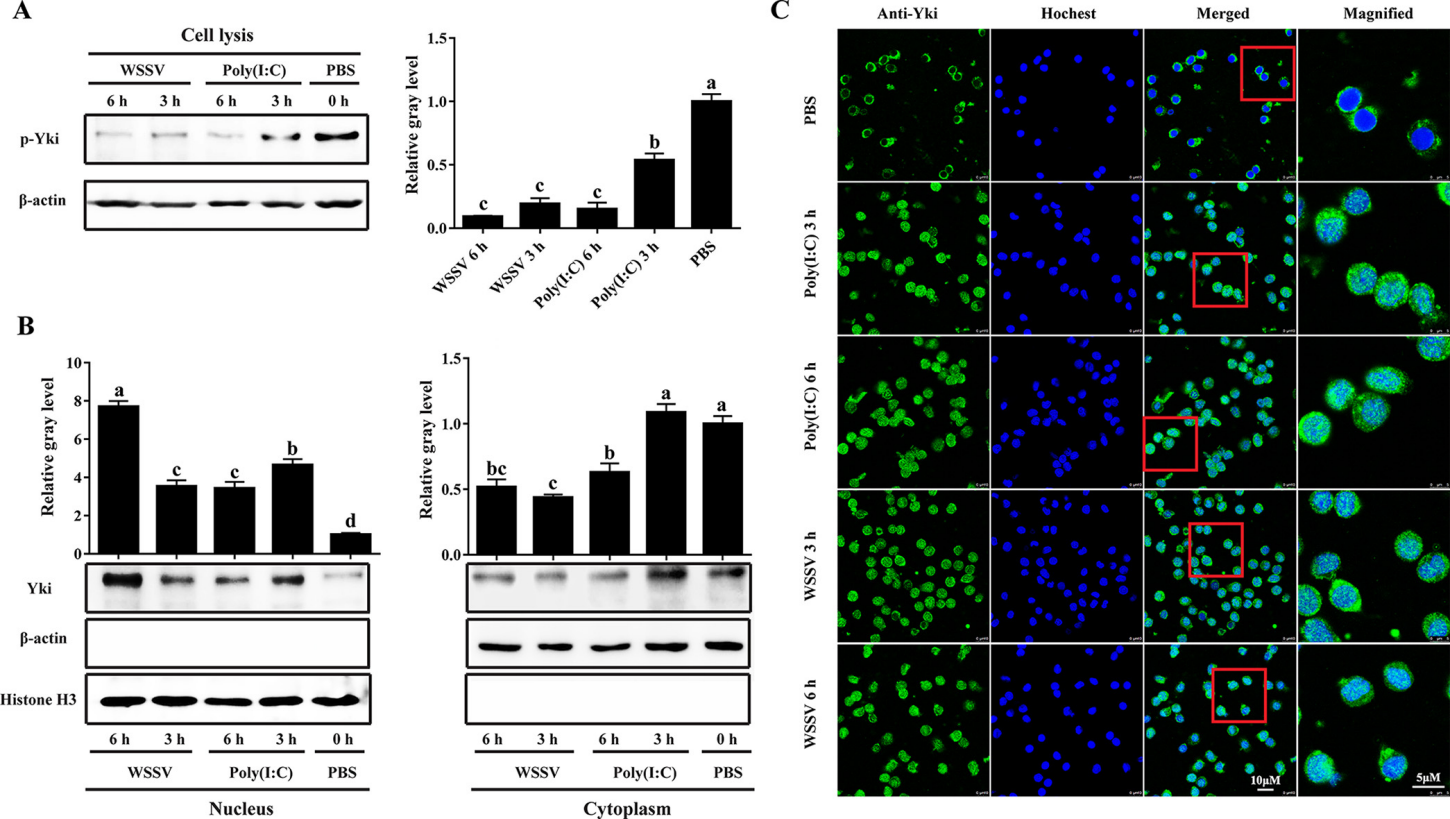
Suppression of Hippo signaling in antiviral immune response. (A) Phosphorylation of Yki in hemocytes after WSSV and poly(I·C) stimulations analyzed by Western blotting. (B and C) Translocation of Yki from nucleus to cytoplasm in hemocytes after WSSV and poly(I·C) stimulations analyzed by Western blotting and immunofluorescence. For Western blotting, the gray values of the Yki and phosphorylated Yki (p-Yki) bands were normalized to those of β-actin or histone H3. **, *P < *0.01 by one way ANOVA with Dunnett’s *post hoc* test.

Activation of Hippo signaling is marked by the phosphorylation and nuclear-translocation inhibition of Yki. The activation state of shrimp Hippo signaling after immune stimulation was therefore investigated. Western blot analysis showed that the phosphorylation of Yki in hemocytes was significantly reduced at 3 and 6 h poststimulation with WSSV and poly(I·C) (Fig. 4A), and correspondingly, the translocation of Yki from cytoplasm to nucleus was increased, especially at 6 h post-WSSV infection, which was 7.7-fold that of the control (Fig. 4B). Consistent with this, the protein level of Yki in cytoplasm was also decreased. The results were further confirmed by immunofluorescence, which demonstrated that WSSV and poly(I·C) promoted the nuclear translocation of Yki (Fig. 4C). This suggests that Hippo signaling is suppressed upon viral infection in shrimp.

### Role of Hippo signaling in WSSV infection.

**FIG 5:**
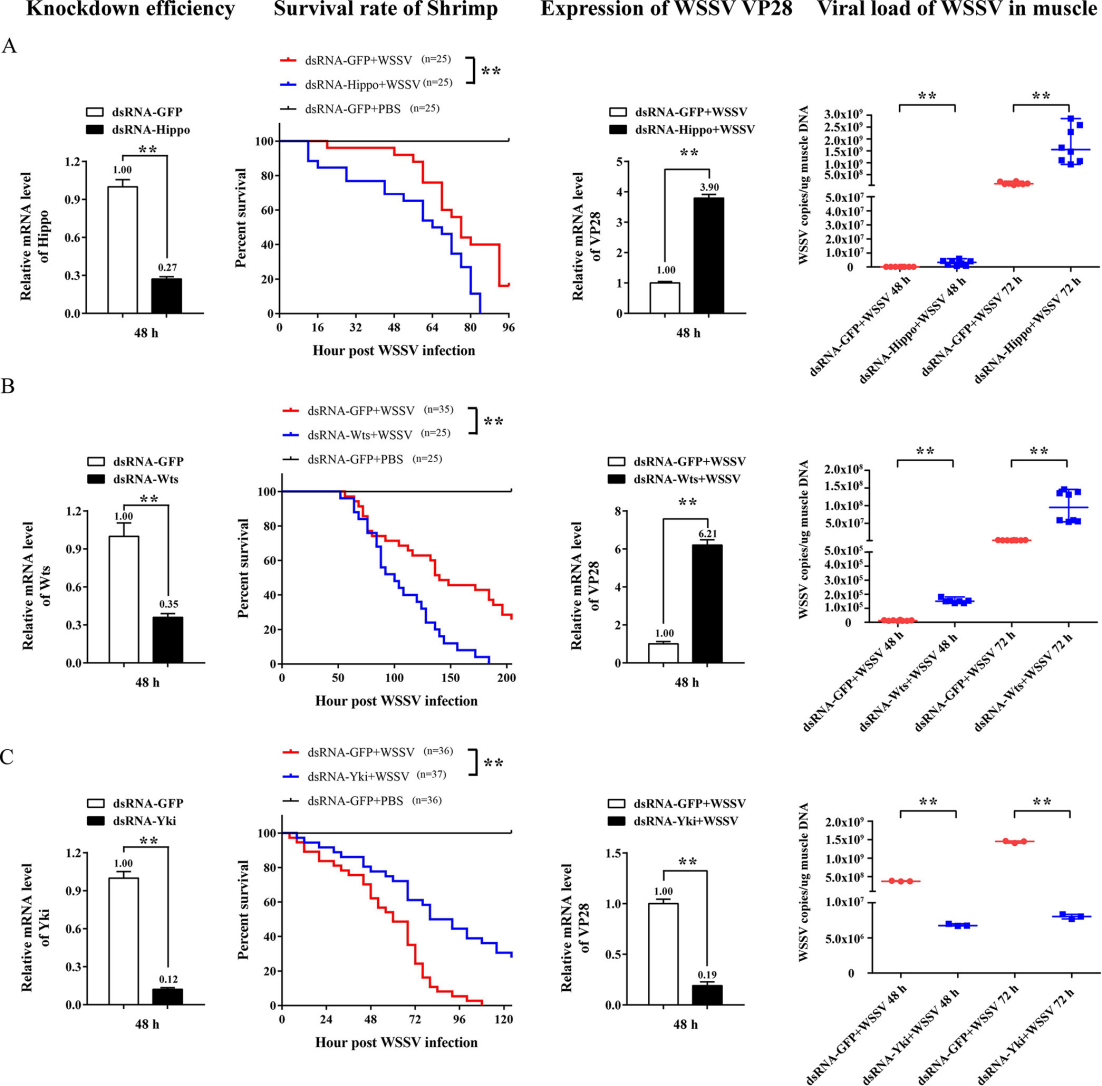
The role of Hippo signaling in WSSV infection. (A to C) After WSSV infection and silencing of *Hippo* (A), *Wts* (B), and *Yki* (C), the mortality of the shrimp was statistically analyzed. **, *P < *0.01 by log-rank (Mantel-Cox) test. The expression of the WSSV *VP28* gene in gill and the copy numbers of WSSV in muscle were examined by qPCR. **, *P < *0.01 by Mann-Whitney test.

To investigate the role of the Hippo pathway in WSSV infection, the RNAi strategy was used to knock down the expression of *Hippo*, *Wts*, and *Yki* before WSSV infection. Compared with the control, after silencing of *Hippo* and *Wts*, the survival rate of WSSV-infected shrimp was significantly decreased, the expression of the WSSV structural gene VP28 in gill at 48 hpi was upregulated, and the viral load of WSSV in muscle at both 48 and 96 hpi was also elevated (Fig. 5A and B). However, after silencing of *Yki*, the survival rate of WSSV-infected shrimp was increased, the expression of *VP28* was downregulated, and the copy number of WSSV in muscle was reduced (Fig. 5C). This indicates that the inhibition of Hippo signaling, i.e., the activation of Yki, facilitated the WSSV infection in shrimp.

### Regulation of hemocyte apoptosis by the Hippo pathway.

**FIG 6:**
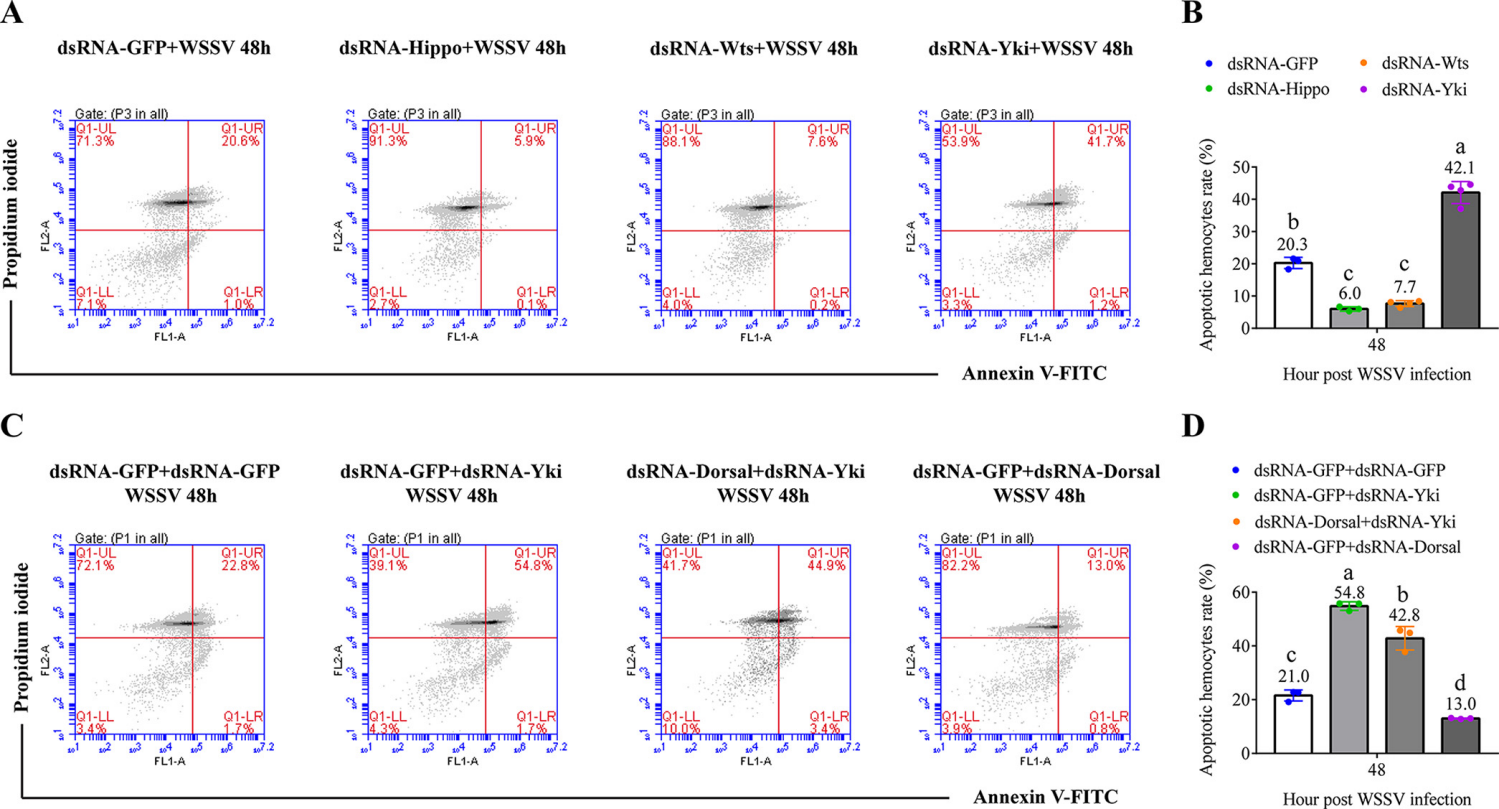
The influence of the Hippo pathway on WSSV-induced apoptosis. (A) The apoptosis of hemocytes in WSSV-infected shrimp after silencing of *Hippo*, *Wts*, and *Yki* by RNAi was analyzed by annexin V-PI staining and flow cytometry; (B) the apoptotic rates were statistically analyzed. (C and D) The apoptosis of hemocytes of WSSV-infected shrimp after cosilencing of *Dorsal* and *Yki*. The dsRNA-GFP was used as a control. *, *P < *0.05; **, *P < *0.01 by one way ANOVA with Dunnett’s *post hoc* test.

Cell apoptosis is an important sign of WSSV infection in shrimp, which contributes to the pathogenesis of WSS. The role of the Hippo signaling pathway in WSSV-induced apoptosis was then explored. We observed that compared with the control, the apoptotic rate of hemocytes from *Hippo*- and *Wts*-silenced shrimp was significantly decreased, while that from *Yki*-silenced shrimp was increased (Fig. 6A and B). To investigate the role of the Yki-Dorsal signaling axis in WSSV-induced apoptosis, hemocytes from *Yki-* and *Dorsal-*specific dsRNA-cotreated shrimp were further analyzed. The results showed that in WSSV-infected shrimp, silencing of *Dorsal* reduced the hemocyte apoptosis compared with the control and attenuated the proapoptotic effect of dsRNA-Yki on hemocytes (Fig. 6C and D).

## DISCUSSION

Although the Hippo pathway has been systemically studied in *Drosophila*, its nature in most other invertebrates remains largely unknown. In the current study, the functions of three key components of the Hippo signaling pathway, Hippo, Wts, and Yki, were investigated in *P. vannamei*. We demonstrated that activation of shrimp Yki, characterized by nuclear translocation, was associated with its dephosphorylation and was negatively controlled by Hippo and Wts. The regulation of Yki activation by these upstream kinases could be abolished by mutation of the phosphorylation site in Yki. Silencing of *Hippo* and *Wts in vivo* promoted the dephosphorylation and nuclear translocation of Yki. These results suggest that the Hippo-Yki pathway in crustaceans operates by the same mechanism as in humans and *Drosophila*, confirming that Hippo signaling is highly conserved across invertebrates and vertebrates.

Accumulating studies have suggested that the Hippo signaling cascade exhibits strong responses to viral infections (28). In mammals, some viruses can affect the components of the Hippo pathway at the expression level. For instance, the molluscum contagiosum virus (MCV) and hepatitis B virus (HBV) upregulate *YAP* (the mammalian homolog of *Yki*) expression in the skin peripheral keratinocytes and hepatocellular carcinoma, respectively (29, 30). In cervical cancer and cervical precancerous lesions, the expression level of *YAP* is also correlated with the integration status of human papillomavirus (HPV) (31, 32). More cases point to the regulation of Hippo signal transduction and YAP activation by viruses in mammals. HPV E6 can promote the nuclear localization of YAP through association with cellular post synaptic density disc-large zo-1 (PDZ) domain proteins (33). Similarly, the vGPCR protein encoded by Kaposi sarcoma-associated herpesvirus (KSHV) facilitates YAP dephosphorylation and nuclear transport by acting through Gq/11 and G12/13 proteins to inhibit Hippo signaling (34). Modulation of Hippo signaling and YAP activation essentially contribute to the pathological process of these virus infections, especially the carcinogenesis of some oncogenic viruses (28, 35). Here, we observed that the expression of all three analyzed Hippo pathway components was upregulated upon WSSV infection and poly(I·C) stimulation. The expression change trends of *Hippo* and *Wts* were similar, suggesting a functional correlation between them. The *Yki* expression increased more significantly after stimulation compared with the two upstream kinases, indicating that Yki tends to be activated upon viral stimulation in shrimp. Furthermore, in hemocytes of the WSSV-infected shrimp, the dephosphorylation and nuclear transport of Yki were significantly enhanced, suggesting an impairment of *Hippo* signaling and an overactivation of Yki after WSSV infection. The suppression of *Hippo* and *Wts* by RNAi facilitated WSSV infection, while the suppression of *Yki* showed opposite results. This indicates that WSSV can block Hippo signaling and activate Yki for favoring its infection in shrimp. On the other hand, the inhibition of Yki by enhancing Hippo signaling may be a strategy for preventing WSSV infection.

Dorsal is one of the only two transcription factor members of the NF-κB family of crustaceans and is important for antiviral responses (26, 36). In static cells, Dorsal is coupled with Cactus, a homolog of the NF-κB inhibitor IκB, to be retained in the cytoplasm. Upon stimulation signals, Cactus is degraded by upstream protease complexes, releasing Dorsal for nuclear translocation to regulate the expression of downstream effector genes (27). In *Drosophila* and oriental river prawn Macrobrachium nipponense, Yki is involved in the induction of Cactus expression and suppresses the activation of NF-κB, which establishes the positive role of Hippo signaling in immunity against bacterial infection (37, 38). In this study, Yki could also enhance the expression of Cactus, and as a result, silencing of *Yki* promoted the nuclear translocation of Dorsal and upregulated the expression of Dorsal-targeted downstream immune effector genes in *P. vannamei*. Consistently, silencing of Hippo produced a result opposite that of Yki. This indicates that the inhibition of Hippo signaling and promotion of Yki activation attenuate the Dorsal pathway in shrimp, thereby weakening the antiviral immunity, which may contribute in part to the promotion of WSSV infection.

As a common pathological phenomenon in host cells after viral infection, apoptosis serves as an essential mechanism of cellular defense to inhibit virus replication and eliminate infected cells (39–41). WSSV infection induces apoptosis in various cell types in crustaceans (42). A recent study suggested that in mud crab Scylla paramamosain, exosomes in the hemolymph could induce hemocyte apoptosis to suppress WSSV replication (43). The current work suggested that the activation of Hippo signaling and blockage of Yki could promote the WSSV-induced hemocyte apoptosis in shrimp. Interestingly, Dorsal was also shown to promote the hemocyte apoptosis after WSSV infection and could offset the antiapoptosis effect of Yki. This suggests that in addition to activating the antiviral immunity of the host, the shrimp Dorsal pathway can also inhibit the replication of WSSV by promoting apoptosis. The activation of Yki upon WSSV infection indicates an excessive inhibition of apoptosis by negatively regulating the activation of Dorsal, which is another mechanism for its facilitating effect on WSSV infection.

The activation of Yki and inhibition of Hippo signaling also occur after poly(I·C) stimulation, indicating that the response of the Hippo pathway may be an active response of host antiviral immunity. Although the current study suggests that activation of Yki promotes WSSV infection, the exact roles of the Hippo pathway in shrimp require further exploration. The nuclear translocation level of Yki after WSSV infection was much higher than that after poly(I·C) stimulation, suggesting that WSSV could reinforce the host response of Hippo signaling. It is possible that WSSV infection may harm the host immune homeostasis, which is not conducive to shrimp antiviral defense. In mammals, the mechanism by which viruses regulate the Hippo pathway is complex. Viruses may present contradictory regulatory effects on Hippo-Yki/YAP signaling. For example, hepatitis C virus (HCV) NS4B impairs Hippo signaling to activate YAP, promoting the migration and invasion of hepatoma cells by activating the downstream PI3K/AKT pathway (44, 45), while the HCV E2 protein enhances the activity of Hippo to suppress YAP activation by mimicking glypican (GPC)-3 binding to CD81, an upstream regulator of the Hippo pathway (46). Therefore, the underlying mechanisms of WSSV-promoted Hippo signaling inhibition and Yki activation in shrimp are worthy of in-depth investigation.

## MATERIALS AND METHODS

### Animal and pathogens.

Healthy Pacific white shrimp (~10 g) were purchased from a shrimp farm in Zhuhai, China. Before experiments, shrimp were acclimated at ~27°C for at least 7 days in a recirculating water tank system filled with air-pumped seawater with salinity of ~1.0%. A random sampling of shrimp (5%) was ensured to be free of Vibrio Parahaemolyticus and *White spot syndrome virus* (WSSV) by PCR using previously reported methods (47, 48). The WSSV stocks were prepared as previously described (49).

### qPCR.

The *P. vannamei Hippo* (XP_027217448), *Wts* (XP_027207893), and *Yki* (XP_027211630) sequences were retrieved from reported genome and transcriptome data (50) and verified by rapid amplification of cDNA ends (RACE) using a SMARTer RACE cDNA amplification kit (Clontech, Japan) and reverse transcriptase PCR (RT-PCR). The primers designed for qPCR are listed in Table 1. For mRNA distribution analysis, shrimp tissues were sampled and pooled from 15 healthy individuals. In the challenge experiments, shrimp were injected with WSSV (10^6^ copies) and poly(I·C) (5 μg) in 50 μL phosphate-buffered saline (PBS). Shrimp injected with PBS were used as controls. Hemocytes pooled from 9 shrimp in each group were sampled at various time points postinjection using acid-citrate dextrose (ACD) anticoagulant (0.48% citric acid, 1.32% sodium citrate, and 1.47% glucose) with a ratio of 3:1 to hemolymph. For analysis of gene expression after double-stranded RNA (dsRNA) treatment, the dsRNA specific to the coding sequences of *Yki* (dsRNA-Yki), *Hippo* (dsRNA-Hippo), *Wts* (dsRNA-Wts), *Dorsal* (dsRNA-Dorsal), and *GFP* (dsRNA-green fluorescent protein [GFP], as the control) were produced using a T7 RiboMAX Express RNA interference (RNAi) system (Promega, USA) following previously described methods (51). Shrimp were injected with dsRNA at 1 μg/g body weight in 50 μL PBS, and 48 h later, hemocytes were sampled and pooled from 9 shrimp. Tissues and hemocytes were treated with RNAlater at 4°C overnight and then stored at −80°C. The RNA extraction, cDNA synthesis, and real-time qPCR analysis were carried out as previously described (52). The qPCR was performed in a 10-μL amplification system containing 5 μL 2× SYBR premix Ex Taq II (TaKaRa, Japan), 500 nM each primer, and 1 μL cDNA on a LightCycler 480 system (Roche, Germany) with previously described parameters (52). Data were calculated using the 2^–ΔΔ^*^CT^* method after normalization to the geometric mean expression of the internal controls GAPDH (glyceraldehyde-3-phosphate dehydrogenase); (GenBank accession no. KT861451) and elongation factor 1 (EF1-α; GU136229) genes.

**TABLE 1:** Summary of primers used in this study

Primers	Sequences (5′ to 3′)
For cDNA cloning	
Hippo-ORF-F	ATGACGAGTCTGGAGAACAAGGAGA
Hippo-ORF-R	GAAATTCTGCTGTCTCTTCCTTTTCTGG
Hippo-5′ RACE1	AGCACCTGCCCAGATTCCT
Hippo-5′ RACE2	GCTTGACCAACTCGCCATT
Hippo-3′ RACE1	AGGTGGAGACGGCACAATG
Hippo-3′ RACE2	CAGCCGAGAATCAACCCAA
Wts-ORF-F	ATGGCCCCTCAGATGCCTCAT
Wts-ORF-R	TACATAGACTGGATGTTGACTGTCCTTC
Wts-5′ RACE1	CCACCAATGCCTGGACTGT
Wts-5′ RACE2	CCCGCAGCATAGGGTAACA
Wts-3′ RACE1	CACCCACCCTGAACAAAGACT
Wts-3′ RACE2	AACGAGAGTGACCTGGAGTGG
Yki-ORF-F	ATGGCCAGCTCGAACAAGGATG
Yki-ORF-R	CAGCCACGTATGGAGATTATCTATTTTGG
Yki-5′ RACE1	GTCGTGGACTGTGTGATGTGG
Yki-5′ RACE2	TTACCCCGTTGCTCCATAGA
Yki-3′ RACE1	AGCAGTCCACACAGCAAACC
Yki-3′ RACE2	CTACCACAGACATCCACACGC
Yki-S112A-F	CACTTCCGGCACCAC GCG TCGCCAGCATCCTTGCAACAGACCT
Yki-S112A-R	CAAGGATGCTGGCGACGCGTGGTGCCGGAAGTGCTGCGGGGCC
RT-PCR analysis	
Hippo-F	GCCCCCTTACGGTGACATT
Hippo-R	GAACTCGGGTTGCCACTGAT
Wts-F	TGGCTCACATACCAATAACTCAA
Wts-R	GGGATGACTCAGACCTGGTGTTA
Yki-F	ACTACCACAGACATCCACACGC
Yki-R	GGGAGTATGAGGGAGGGAGTAA
Cactus-F	GGAGGCGTGCCAGTGACTATG
Cactus-R	GAAGTAACGATCTGCATTGAAGGG
Dorsal-F	TTGCGACCACCAGACAAGAG
Dorsal-R	GCAAGGTAACGACTAATCTTCTCTG
ALF4-F	CCTGGTGGCACTCTTCGC
ALF4-R	ACGGTGAAGCGGCACTTATG
LYZ1-F	TACGCGACCGATTACTGGCTAC
LYZ1-R	AGTCTTTGCTGCGACCACATTC
PEN3-F	CTCCTGCGTCCGCCATG
PEN3-R	GTGTAACCGCCCTTGTACAC
Cru2-F	GGTACGTCTGCTGCAAGCC
Cru2-R	CTGAGAACCTGCCACGATGG
EF-1α-F	CCTATGTGCGTGGAGACCTTC
EF-1α-R	GCCAGATTGATCCTTCTTGTTGAC
GAPDH-F	TCAACGAGATGAAGCCCGA
GAPDH-R	CACCAGTGGACTCAACGATGTA
VP28-F	TGAGGTTGGATCAGGCTACTTC
VP28-R	CCGCATCTTCTTCCTTCATCTG
WSSV32678-F	GTTTTCTGTATGTAATGCGTGTAGG
WSSV32753-R	CCCACTCCATGGCCTTCA
TaqMan probe-WSSV32706	CAAGTACCCAGGCCCAGTGTCATACGTT
dsRNA production	
dsHippo-F	GGCAGAACCAAATCAAGCATA
dsHippo-R	TCTTCTAAAGGCAACTGGGATG
dsHippo-T7-F	GGATCCTAATACGACTCACTATAGG GGCAGAACCAAATCAAGCATA
dsHippo-T7-R	GGATCCTAATACGACTCACTATAGG TCTTCTAAAGGCAACTGGGATG
dsWts-F	AGTGAAGTAGTGCCAACCACAGAT
dsWts-R	GAGATGATGTGGCAGTAGAAAGG
dsWts-T7-F	GGATCCTAATACGACTCACTATAGG AGTGAAGTAGTGCCAACCACAGAT
dsWts-T7-R	GGATCCTAATACGACTCACTATAGG GAGATGATGTGGCAGTAGAAAGG
dsYki-F	ACGCAAGTGAGCCACCAGT
dsYki-R	TCCGTGGACAGGTCATCATT
dsYki-T7-F	GGATCCTAATACGACTCACTATAGG ACGCAAGTGAGCCACCAGT
dsYki-T7-R	GGATCCTAATACGACTCACTATAGG TCCGTGGACAGGTCATCATT
dsDorsal-F	TGTATCTCTTCGGAGGTTGGAC
dsDorsal-R	AACATTGTGCTGGGCTGACT
dsDorsal-T7-F	GGATCCTAATACGACTCACTATAGGTGTATCTCTTCGGAGGTTGGAC
dsDorsal-T7-R	GGATCCTAATACGACTCACTATAGGAACATTGTGCTGGGCTGACT
GFP-F	ATGGTGAGCAAGGGCGAGGAG
GFP-R	TTACTTGTACAGCTCGTCCATGCC
T7-GFP-F	GGATCCTAATACGACTCACTATAGGATGGTGAGCAAGGGCGAGGAG
T7-GFP-R	GGATCCTAATACGACTCACTATAGGTTACTTGTACAGCTCGTCCATGCC

### Shrimp challenge.

At 48 h post-dsRNA injection, shrimp were challenged with 10^6^ copies of WSSV and divided into two groups, one to record mortality and the other to sample tissues at 48 hpi. The viral load of WSSV in muscle and *VP28* expression in gill were determined by absolute qPCR and RT-qPCR as previously described (53). Experiments were done in triplicate using different batches of shrimp, and the results with similar trends were representatively shown.

### Immunofluorescence.

Hemolymph was extracted as described above. After washing and resuspension with normal saline, hemocytes were placed on silicified glass slides for 30 min for adhesion. Hemolymph smears were fixed with 4% paraformaldehyde, permeabilized with 1% Triton X-100, and blocked with 10% normal goat serum. After incubation with rabbit antibodies against shrimp Dorsal or Yki (GL Biochem, China), hemocyte smears were detected using Alexa Fluor 488-conjugated goat anti-rabbit IgG antibody (Abcam, USA), followed by staining with Hoechst 33342 (Sigma, USA). Sections were observed with a Leica LSM 410 confocal microscope (Germany).

### S2 cell visualization.

The serine at position 112 of Yki was changed to alanine by overlapping PCR to generate the Yki-S112A mutant. The open reading frames (ORFs) of *P. vannamei Hippo*, *Yki*, and *Yki-S112A* were cloned into the pAc5.1-GFP vector (54) to express GFP-tagged proteins. The *Wts* ORF with a hemagglutinin (HA) tag-encoding sequence was cloned to the pAc5.1/V5-HisA vector (Invitrogen, USA). *Drosophila* S2 cells at ~80% confluence were transfected or cotransfected with these vectors using FuGENE HD transfection reagent (Promega, USA). At 48 h posttransfection, the expression of Wts-HA was analyzed by immunofluorescence using mouse anti-HA antibody (Cell Signaling Technology [CST], USA) and Alexa Fluor 594-conjugated goat anti-mouse IgG antibody (Abcam, USA). After staining with Hoechst 33342, cells were observed using a confocal laser scanning microscopy at 350/488 nm excitation wavelengths.

### Western blot analysis.

To analyze the phosphorylation of Yki, hemocytes extracted from shrimp were directly treated with 1× SDS sample buffer, separated on 12% SDS-PAGE, and transferred onto polyvinylidene difluoride (PVDF) membranes. After blocking with 10% skim milk powder in PBS-T buffer (PBS with 0.5% Tween 20), the blotting membranes were analyzed using a rabbit anti-phosphor-YAP (S127) antibody (Cell Signaling Technology, USA) for phosphorylated Yki and an anti-β-actin antibody (MBL, Japan) for the internal control. To analyze the nuclear translocation of Yki, the cytoplasmic and nuclear proteins were separated using NE-PER nuclear extraction reagents (Thermo, USA). Western blotting was performed using the rabbit antibody against shrimp Yki (GL Biochem, China), the anti-β-actin antibody, and the anti-histone H3 antibody (CST) (for the internal nuclear control). After incubation with the appropriate secondary antibodies, the membranes were developed by chemiluminescence using a Pierce ECL Western blotting substrate (USA). The gray values of specific protein bands were analyzed using the Gauss model on Quantity One 4.6.2 software (Bio-Rad, USA), which were further normalized to those of the internal control proteins as previously described (54).

### Apoptosis analysis.

Shrimp were treated with dsRNA and 48 h later were further challenged with WSSV. Following the instructions of an annexin V-fluorescein isothiocyanate (FITC)/propidium iodide (PI) apoptosis detection kit (Sigma-Aldrich, USA), hemocytes were sampled at 48 hpi and resuspended in 1× Annexin V binding buffer at a concentration of 1 × 10^6^/mL, stained with 5 μL annexin V-fluorescein isothiocyanate (FITC) and 10 μL PI for 10 min in dark, and detected using a BD Accuri C6 flow cytometry (USA).

### Statistical analysis.

Experiments were replicated three times using different batches of shrimp, and data were representatively shown. Statistical comparisons were performed by Mann-Whitney test or one-way analysis of variance (ANOVA) followed by Dunnett’s *post hoc* test using SPSS software. Data are presented as the mean ± standard deviation (SD). The log-rank (Mantel-Cox) test was used to analyze the survival rates.
